# Electrospinning of Cyclodextrin Functional Nanofibers for Drug Delivery Applications

**DOI:** 10.3390/pharmaceutics11010006

**Published:** 2018-12-24

**Authors:** Fuat Topuz, Tamer Uyar

**Affiliations:** Institute of Materials Science & Nanotechnology, UNAM-National Nanotechnology Research Center, Bilkent University, 06800 Ankara, Turkey

**Keywords:** cyclodextrin, electrospinning, drug delivery, nanofibers, cyclodextrin-inclusion complexes, essential oils, electrospun nanofibers, poly-cyclodextrin, antibacterial, antibiotics

## Abstract

Electrospun nanofibers have sparked tremendous attention in drug delivery since they can offer high specific surface area, tailored release of drugs, controlled surface chemistry for preferred protein adsorption, and tunable porosity. Several functional motifs were incorporated into electrospun nanofibers to greatly expand their drug loading capacity or to provide the sustained release of the embedded drug molecules. In this regard, cyclodextrins (CyD) are considered as ideal drug carrier molecules as they are natural, edible, and biocompatible compounds with a truncated cone-shape with a relatively hydrophobic cavity interior for complexation with hydrophobic drugs and a hydrophilic exterior to increase the water-solubility of drugs. Further, the formation of CyD-drug inclusion complexes can protect drug molecules from physiological degradation, or elimination and thus increases the stability and bioavailability of drugs, of which the release takes place with time, accompanied by fiber degradation. In this review, we summarize studies related to CyD-functional electrospun nanofibers for drug delivery applications. The review begins with an introductory description of electrospinning; the structure, properties, and toxicology of CyD; and CyD-drug complexation. Thereafter, the release of various drug molecules from CyD-functional electrospun nanofibers is provided in subsequent sections. The review concludes with a summary and outlook on material strategies.

## 1. Introduction

Electrospun nanofibers of synthetic, natural, and hybrid systems have been widely exploited as drug delivery materials due to their high specific surface area, which allows enhanced drug loading and ability to modulate release profiles by structural tuning [1,2,3,4,5,6,7,8,9,10,11]. They can be engineered in various shapes, textures, and sizes with diameters down to a few nanometers. Further, the fiber surface can be modified with specific functional ligands or molecules to hinder them from the nonspecific adsorption of proteins or make them cell adhesive via decoration with cell binding ligands [3,12,13]. Drugs can be loaded into electrospun nanofibers by blending prior to electrospinning or using specific chemistry for the controlled release of drugs from the fiber matrix [14,15]. Although there are many pros of electrospun nanofibers for drug delivery applications, they cannot serve as injectable drug reservoirs. On the other hand, the drug-loaded nanowebs or fiber-deposited meshes are quite promising as wound dressing materials [11,12]. Apart from their physical protection to the injured tissue, they can provide the sustained release of the entrapped drugs to achieve wound debridement and wound healing simultaneously [16]. In this regard, comprehensive reviews on the use of electrospun nanofibers for wound healing are available [16,17]. Likewise, the use of CyD-based hydrogel materials for wound dressing has been reviewed in detail [18]. As most drugs are hydrophobic compounds and thus not intrinsically water soluble, the incorporation of a considerable amount of drug molecules into electrospun nanofibers while maintaining their activity can be problematic. In this context, the use of CyD enhances the solubility of the embedded drugs while keeping them stable and bioavailable for enhanced therapy results. The drug release can take place as CyD/drug ICs, if CyD are not chemically attached to the material, or by altering the surrounding conditions that lead to entropically unfavorable inclusion complexation. 

The use of drug-loaded electrospun mats as implant materials has taken considerable interest in wound healing. The release of the embedded drugs can take place with the degradation of nanofibers. With that, the entrapped drug molecules can be released from the fiber matrix. Particularly, the use of some specific drugs can accelerate wound healing process and reduce pain. Furthermore, the drug release can be tuned by the structure of the fiber components: sustained drug release can be obtained for the electrospun fibers made by hydrophobic polymers or structurally tuned fiber systems. In one example, core-sheath structured nanofibers with core-loading of hydroxycamptothecin (HCPT) were used on mice via intratumoral implantation [19]. The use of hydroxypropyl β-CyD (HP-β-CyD) molecules as an additive significantly fastened the HCPT release and allowed the higher degradation of emulsion electrospun fibers. The higher release of the loaded HCPT was ascribed to the distribution pattern of HP-β-CyD and HCPT within the fibers. In another example, CyD inclusion complexes (CyD:ICs) with perfluoroperhydrophenanthrene (PFP) as oxygen carriers to cells seeded on the electrospun scaffolds of poly(carbonate urethane) (PCU) and polycaprolactone (PCL) [20]. The ICs of PFP and CyD significantly increased the amount of the dissolved oxygen. Such a concept can be exploited in in vivo applications to fasten the healing of wounded tissues. 

In the electrospinning, the texture, size, and structure of nanofibers can be tailored over electrospinning parameters and polymer formulation [21]. Such control on the fiber structure endows them with enhanced performance in drug loading and allows the sustained release of the embedded drug molecules. In this regard, several polymer-based fiber systems were implemented in drug delivery applications [1,22]. Particularly, biocompatible polymers are preferably chosen since they do not release any toxic products during their use. In this regard, polycaprolactone (PCL), poly-l-lactic acid (PLLA), and polycaprolactone/poly(ethylene oxide) (PCL/PEO) are the most widely used polymeric materials [23,24,25,26]. The degradation of such nanofibers takes place over hydrolysis, starting from the surface to the core through surface erosion without leaving any toxic byproducts [27]. Along with the fiber degradation, the entrapped drug molecules are released from the fiber matrix. The sustained drug release from such polymers is greatly influenced by the structural features of polymers (e.g., hydrophobicity and glass-transition temperature (*T*_g_)) and the type of the used spinneret system. In this context, a comprehensive review on the sustained release of drugs from electrospun nanofibers was reported by Chou et al., in which the release from uniaxial and coaxial nanofibers from various polymers was deliberately discussed [28]. When hydrophilic electrospun nanofibers are used, the degradation mostly follows bulk erosion with the breakage of hydrolytically labile bonds. In other words, the bulk erosion takes place once the water diffusion is much faster than the scaffold degradation, followed by mass loss throughout the bulk of the material [29].

The crucial problems in drug delivery applications are (i) uncontrolled release of drug molecules (i.e., burst release), (ii) unsustainable drug delivery, (iii) low drug loading, and (iv) low stability and bioavailability of drugs. In this regard, the electrospinning can minimize these problems to some extent because of its unique features, including easy to use with tailored-made fiber properties and applicability to a wide range of materials, such as polymers, composites, and ceramics with fiber sizes ranging from nanometer to micrometer. Further, the functionalization of electrospun nanofibers with pharmaceutical excipients, such as CyD, improves their performance in drug delivery applications. CyD comprise unique features that are often desired in drug delivery carriers. For instance, drug molecules can be entrapped into the hydrophobic molecular-environment of CyD by inclusion-complexation, which dramatically increases the stability of drug molecules under harsh conditions, e.g., high temperature, sunlight, and pH [30]. Further, such complexation remarkably increases their water solubility since many drugs are hydrophobic molecules. Most importantly, for a drug molecule to be pharmacologically active, it should have significant water solubility and lipophilicity to be able to permeate biological membranes through passive diffusion so that no accumulation occurs that can give rise to toxicity. In this regard, lipophilic CyD may assist them in crossing the biological membranes through component extraction or fluidization and can minimize the immunogenic response of body [31]. Further, the surface of the electrospun nanofibers becomes crucial for their in vivo applications since the biocompatibility of the nanofibers and their interactions with the immune system are greatly defined by their surface chemistry. Undesired protein adsorption may occur rapidly when the material is implanted. The adsorbed proteins can denature on hydrophobic surfaces and thus affect the immune system and wound healing. Hence, the surface chemistry becomes a highly critical factor when the fiber mats are intended to be used in in vivo drug delivery.

CyD-functional electrospun nanofibers have been engineered using different approaches. Although most research has been focused on CyD/drug inclusion-complexes (ICs)-embedded polymeric electrospun nanofibers, the recent decade has witnessed significant advances in the polymer-free electrospinning of CyD and their use in drug delivery applications. Functional electrospun nanofibers were also produced using poly-cyclodextrin (polyCyD) molecules in the fiber matrix. In this regard, various active agents either with anticancer, antibacterial, antioxidant or anthelmintic properties have been incorporated into such nanofibers and exploited in drug delivery systems. In the first part of this review, some of the intriguing features of CyD and the mechanism of inclusion-complexation and drug solubility/stability are given. Afterward, several approaches based on CyD-functional nanofibers for drug delivery applications are discussed. The review ends with a future outlook and concluding remarks.

## 2. Electrospinning

Electrospinning is a versatile process that relies on the jetting of a viscous solution or polymer melt under an electrical field [32]. With the evaporation of solvent molecules, the solidified jet is directed to a collector by electrical forces. During the electrospinning, the fiber is subjected to many different forces, including aerodynamic, inertial, gravitational, rheological, and tensile forces [33,34]. Once an electrical voltage is applied, free charges in the solution lead to the movement and rapidly transfer a force to the electrospinning solution to flow. In this regard, the surface tension, viscoelasticity, and charge density of electrospinning solutions are the key factors for the formation of electrospun nanofibers. The texture, morphology, and size of the nanofibers depend on the processing parameters and solution properties. Mostly, polymeric solutions were used to produce continuous electrospun fibers due to their high viscosity and the presence of intra- and intermolecular interactions. Sometimes, because of the instability of the jet of polymer solutions, the formation of beaded fibers can be observed [35,36,37]. However, with increasing viscosity or tailoring other solution parameters, bead-free electrospun nanofibers can be obtained [37]. Further, various additives can be incorporated in the electrospinning solution to produce functional nanofibers.

**Figure 1:**
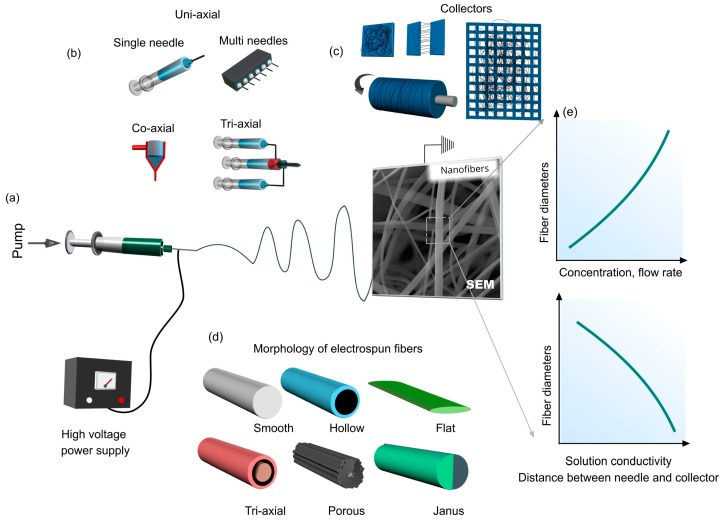
An electrospinning setup with important parameters is shown. (**a**) A cartoon scheme of an electrospinning system with the scanning electron micrograph of electrospun fibers, (**b**) common spinneret systems used in electrospinning, (**c**) collector types, (**d**) the morphology of electrospun fibers, and (**e**) diagrams showing the influence of electrospinning process parameters and solution properties on the electrospun fibers.

The fiber stability can be provided using either hydrophobic molecules or cross-linking approaches to attain water-insoluble fiber meshes. The main advantages of the electrospinning process are as follows: (i) easy to operate, (ii) adaptability to various polymeric systems, and (iii) suitability to prepare the nanofibers with various diameters, textures, and structures. Figure 1 shows a general representation of an electrospinning setup together with some important processing parameters and nanofibers in various forms. The presence of additives can lead to nanofibers with different morphologies. Various nanoparticles can be loaded in the electrospinning solutions by blending to yield hybrid fibers, or the thermal treatment of nanofibers impregnated with inorganic or metallic precursors. Using different spinneret systems, hollow, core-shell, and triaxial fiber structures can be obtained. Particularly, the co-axial fibers show better sustained release profiles than uniaxial fiber systems as the outer shell acts as a molecular gate in the transport of drug molecules. The fiber size can simply be adapted by the electrospinning parameters or solution conductivity while the solution properties allow the fabrication of ultrafine fibers having diameters in the nanoscale with different morphologies and textures (Figure 1). In general, increasing the polymer concentration, and flow rate causes the formation of larger nanofibers [38]. On the other hand, the fiber diameter decreases with increasing distance between the needle and collector and solution conductivity [38]. In this regard, the addition of salts is a common approach to produce thinner nanofibers for various polymeric systems [39]. 

## 3. Cyclodextrins

Cyclodextrins (CyD) are cyclic oligosaccharides of glucopyranose formed during enzyme-catalyzed degradation of starch by glucosyltransferase over chain splitting and intramolecular rearrangement [40]. The molecular structure of CyD resembles a torus-like molecular ring, of which the interior is partially hydrophobic, while the exterior is hydrophilic because of many hydroxyl groups [41]. The inner cavity of CyD accommodates small hydrophobic molecules or portions of large compounds that can fit into their cavities [42]. Owing to their complexation with a wide spectrum of lipophilic molecules, CyD have been implemented in diverse applications, including solubilization enhancers, drug delivery, textile and food industry, tissue engineering, and allied applications [41]. 

CyD molecules do not elicit an immune response, have low toxicity, and hence are extensively used in bio-related fields, particularly to improve the bioavailability of drugs. In the following section, brief information is provided on the structure, properties, and toxicology of CyD and their inclusion-complexation with guest molecules. 

### 3.1. Structure and Properties of Cyclodextrins 

**Figure 2:**
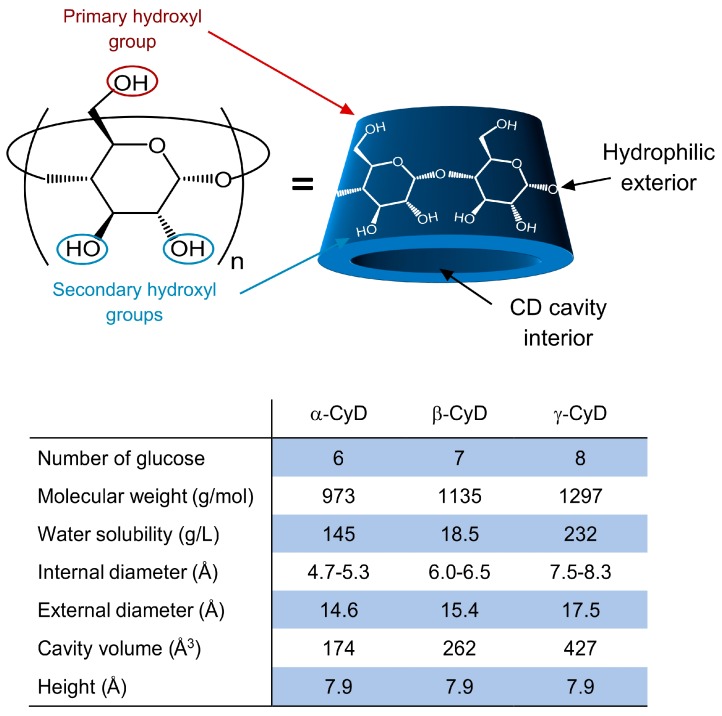
The chemical structure and the representative cartoon illustration of a native cyclodextrin (CyD) molecule in the 3D form. The general characteristics of CyD are given in the inset table [49].

Due to the ^4^C_1_ chair conformation (all equatorial groups in ^4^C_1_ become axial and vice versa) of each glucopyranose unit, CyD have a shape of a hollow truncated cone, of which the cavity interior has a partial hydrophobic character because of hydrogen atoms of C-3, C-5, and the oxygen atoms of the glycosidic link [43]. Whereas the exterior of CyD is hydrophilic owing to the presence of several hydroxyl groups. Although the existence of numerous CyD with various ring sizes in the class of cycloamyloses, the most common ones are glucose hexamer, heptamer and octamer, which respectively have 6, 7, and 8 glucopyranose units (i.e., α-CyD, β-CyD, and γ-CyD) (Figure 2). The first reference to CyD was published in 1891 as the byproduct of bacterial digestion of starch, and at that time it was named as cellulosine [44]. After the exploration of their 3D structure in 1942 by X-ray analysis, they have been considered as complex-forming molecules, and to date, CyD have become common excipients for a diverse range of applications, including pharmaceuticals, foods, agrochemicals, and fragrances [45,46,47,48]. Figure 2 shows the molecular structure of a native CyD molecule. The interior size rises from ~5 to ~8 Å with increasing glucopyranose units from 6 to 8. Even though all CyD have an identical cavity height of ~7.9 Å, the cavity volume shows significant variations from 174 to 427 Å^3^ with increasing the number of glucopyranose units from 6 to 8. 

Due to intermolecular hydrogen bonds, native CyD are not very water-soluble compounds. Particularly, β-CyD molecule has very limited water solubility (~18.5 g/L) while α-CyD and γ-CyD molecules have better water solubility at 145 and 232 g/L, respectively. The poor solubility of β-CyD is originated by the formation of a hydrogen-bond network between the secondary hydroxyl groups [50]. To enhance the water-solubility of the native CyD, they have been chemically modified with different functional groups via amination, esterification, or etherification to break 2-OH-3-OH hydrogen bonds. This also causes the loss of crystallization due to the formation of a statistically substituted material that is made up of many isomeric components with the resultant amorphous, highly soluble end-product. The relatively hydrophobic character of the cavity interior makes CyD ideal molecular carriers of numerous hydrophobic molecules, whose size should be small enough to fit into the cavity. In this context, CyD can form host-guest complexes with a diverse range of organic molecules, inorganic ions, rare gases, and coordination compounds [51,52,53,54]. For interested readers, comprehensive reviews on all aspects of CyD and their applications in drug delivery are available [40,55,56].

### 3.2. Toxicological Issues of Cyclodextrins

For their bio-related applications, CyD must possess some critical conditions, such as biocompatibility and biodegradation. CyD are biocompatible pharmaceutical excipients and have found a wide spectrum of biological use. Even though they have many applications, there are some critical remarks that must be taken into account in their in vivo use. CyD are relatively stable molecules against degradation by human enzymes, and, in this regard, it was reported that after intravenous uptake of CyD by humans, they are excreted intact via the kidney. On the other hand, CyD can be degraded by bacterial and fungal enzymes (i.e., amylases), and hence, in the body, CyD are metabolized in the colon before excretion. The toxicity of CyD arises from their administration route; for instance, for mice exposed to CyD intravenously, the dose that causes 50% death (LD_50_) is 1.0 g/kg, 0.79 g/kg, and more than 4.0 g/kg for α-CyD, β-CyD, and γ-CyD, respectively [57,58]. Particularly, the uptake of high β-CyD content caused toxicity because of its low water solubility (i.e., 18.5 g/L) [59]. The poor water solubility of β-CyD led to microcrystalline precipitation in the kidney. Further, β-CyD altered the cell membrane permeability by leading hemolysis because of the binding and extraction of cholesterol through inclusion-complexation [60]. Likewise, Zimmer et al. reported that HP-β-CyD interact with cholesterol crystals and dissolve them, which enhance oxysterol production and promote the anti-inflammatory reprogramming of macrophages [61]. Moreover, β-CyD damage renal cells by the extraction of cholesterol from kidney membrane and lead to nephrotoxicity [62]. In vivo studies showed the insignificant amount of CyD adsorbed from the intestinal tract in intact form. The major part of orally administered CyD is metabolized in the colon, and the primary metabolites are further metabolized to CO_2_ and H_2_O. Because of structural differences between native CyD and CyD derivatives, the adsorption, distribution, and excretion of CyD might have different profiles. Even though the oral administration of CyD did not reveal any significant toxicity, some studies reported their adverse effects on long-term parenteral administration. In this regard, Kantner et al. reported subcutaneous long-term administration of HP-β-CyD with a daily dose of 200 mg/kg resulted in increased bone resorption and bone loss [63]. Even at low dosages (50 mg/kg), minor changes in bone metabolism were also observed. The 2-hydroxypropylation of β-CyD minimizes these toxic effects due to high water solubility of the resultant compound with the condition of less than 1.5% unmodified β-CyD presence. The native β-CyD have been approved in the USA as Generally Recognized as Safe (GRAS) [64]. Likewise, the modified CyD, particularly hydroxypropyl (HP) β-CyD and sulfobutyl ether (SBE) β-CyD, are also included in the FDA (Food and Drug Administration) list as approved chemicals for human use [65]. The metabolism of CyD inclusion complexes (ICs) on oral administration of CyD-ICs, they rapidly dissociate, and the guest molecule leaves the cavity [66]. Thereafter, CyD and guest molecules are involved in the normal biological pathway to be metabolized and excreted from the body. Overall, CyD are generally non-toxic chemicals and can potentially be used for many different biological applications. 

### 3.3. Mechanism of Cyclodextrin Inclusion-Complexation and Drug Solubility

**Figure 3:**
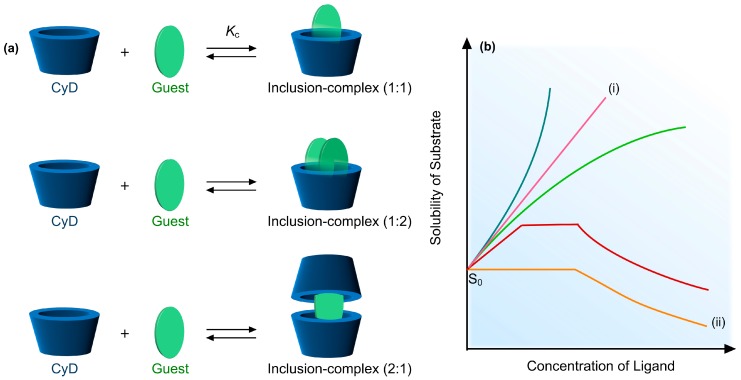
(**a**) The inclusion complex formation between CyD and guest molecules at various stoichiometries. (**b**) The plot shows a phase solubility of guest molecules; (i) represents the formation of soluble inclusion-complex (IC), and (ii) denotes the formation of IC with limited solubility.

Many drug molecules are poorly soluble molecules in water and, hence, have affinity to complex with CyD. As the inner cavity of CyD molecules has partial hydrophobic character, they can accommodate small lipophilic molecules into their cavities and significantly enhance their water-solubility via inclusion-complexation. The main driving force of the inclusion-complexation relies on hydrophobic interactions between guest molecules and the CyD cavity. Further, other forces, such as van der Waals and dipole-dipole interactions may also be involved in the inclusion-complexation [67,68]. The outer surface of CyD forms hydrogen bonds with water to make them water-soluble. In the inclusion-complexation, intermolecular interactions occur between CyD and guest molecules as partial or the complete penetration of a guest molecule into the CyD cavity. Figure 3a depicts a guest molecule entrapped in the CyD cavity, driven by inclusion-complexation. The inclusion-complexation may also occur in diverse ways as shown in Figure 3a. Depending on the conditions, one guest molecule can complex with two CyD molecules or vice versa (i.e., two guest molecules with one CyD). The interaction between CyD and guest molecule is in an equilibrium directed by an equilibrium constant (*K*_c_). Figure 3b displays the phase solubility plot for guest molecules, where the increased solubility is associated with the CyD concentration. Each line highlights the type of inclusion-complex (IC) formed, as well as its stoichiometry. Linear line (shown in pink color (i)) represents the formation of soluble IC, while the line in orange color (ii) depicts the formation of IC with limited solubility.

Many studies have reported that the inclusion-complexation is governed by van der Waals, electrostatic forces, hydrophobic interactions, hydrogen bonding, and the release of conformational strain [43,69,70,71]. Some parameters define the strength and relative stability of the complexation. Particularly, the size of cavity and guest molecule, the presence of modified groups in the CyD structure, polarity, and substitution groups on the guest molecule, and environmental conditions, such as medium, ionic strength, and temperature, are prominent factors that can affect the relative strength of inclusion-complexation [72,73]. Normally, a CyD molecule exists in a hydrated form in water—that is, CyD cavity accommodates many water molecules. In the presence of a guest molecule, the replacement between high-energy water molecules and a hydrophobic guest is favored because of an energetically unfavored state of water molecules in the hydrophobic cavity, [41,74]. In general, α-CyD forms complexes with aliphatic chains and molecules (e.g., PCL and poly(ethylene glycol) (PEG)) [75,76,77], whereas larger cavity of the β-CyD allows host-guest complexes with aromatic rings, such as polycyclic aromatic hydrocarbons (PAHs) [78,79] and essential oils [80,81].

### 3.4. Drug Stability and Release from Cyclodextrin Inclusion Complexes

One of the currently important issues in drug delivery is the stability of drug molecules on exposure to some harsh conditions. Particularly, biological drugs are sensitive because of their limited stability upon oral administration and during subsequent circulation. They have low bioavailability and short therapeutic half-lives [82]. In this regard, the CyD cavity is enrolled as a shield to protect them from degradation and increase their bioavailability. CyD are stable molecules and can maintain their torus-like molecular structure even at basic pH values [83]. Further, the pyrolysis of CyD molecules starts over 300 °C, implying their thermal stability at high temperature conditions [84]. The inner cavity of CyD molecules accommodates various hydrophobic small molecules and increases their thermal stability. In this regard, various volatile substances (e.g., essential oils) were treated with CyD molecules to form inclusion-complexes for extending their shelf-lives [85,86]. Major benefits of CyD in their drug delivery are (i) increasing the water solubility, stability and bioavailability, (ii) reducing the evaporation of volatile active agents, (iii) low degree of hemolysis, and (iv) avoiding admixture incompatibilities [50].

Hydrophobic and van der Waals interactions are the main dominant driving forces for inclusion- complexation [87]. Therefore, the stability of CyD-ICs is highly dependent on the surrounding conditions that influence these interactions: if pH, ionic strength and temperature of the medium change, guest molecules may leave the CyD cavity because of an energetically unfavored state [72]. This can also be achieved by altering other conditions, making CyD-IC thermodynamically unfavorable. 

## 4. Cyclodextrin Functional Electrospun Nanofibers for Drug Delivery Systems

Various CyD-functional electrospun nanofiber-based materials have been reported as drug delivery systems. These include the blending of polymers with CyD/drug ICs and using CyD-based polymers with drugs. Further, the electrospun nanofibers of only CyD/drug ICs were also performed without the requirement of a polymeric carrier. Due to the absence of a polymeric component, such nanostructured materials present high loading of active agents complexed with CyD for their use in drug delivery. In the following sections, we subcategorize CyD-functional electrospun nanofibers by means of their route of preparation.

### 4.1. Cyclodextrin-Drug Encapsulated Electrospun Polymeric Nanofibers for Drug Delivery Applications

This is the simplest method to engineer drug-encapsulated electrospun nanofibers through the blending of components (i.e., CyD, drug and polymeric matrix). Generally, CyD and drug are mixed to form inclusion complexes (ICs), and then, blended with the polymer solution, of which the electrospinning produces drug-encapsulated nanofibers. The inclusion-complexation with CyD motifs significantly enhances the water solubility of drug molecules so that high loading capacities can be achieved. In this regard, the Uyar research group reported naproxen (NAP)-CyD ICs loaded PCL nanofibers [88]. Naproxen (NAP), a non-steroidal anti-inflammatory drug, is used in the treatment of pain, inflammation, and fever [89,90]. Due to its lipophilic nature, it is practically insoluble in water at low pH, while it becomes soluble at high pH. To make them water-soluble under mild conditions, their ICs were prepared by slowly adding β-CyD into the aqueous solution of NAP, and the solution was stirred overnight in water until it became cloudy. Thereafter, the solution was freeze-dried, and IC powder was obtained. The complexation between CyD and NAP significantly enhanced the water solubility of NAP. The ICs were mixed with PCL solution and electrospun to form nanofibers. The mean diameter of PCL nanofibers slightly increased from ~335 to ~390 nm with the incorporation of NAP-β-CyD ICs. The release studies showed enhanced release of NAP from NAP-β-CyD ICs encapsulated PCL nanofibers when compared to the pristine NAP-loaded PCL nanofibers prepared in the absence of CyD. Likewise, Akduman et al. reported naproxen-CyD embedded polyurethane nanofibers and observed that the complete release of NAP from the only polyurethane fiber matrix took 120 h while the use of β-CyD or HP-β-CyD reduced the delivery time for NAP to ~36 and 5 h, respectively [91]. The difference in the release profiles from both CyD was due to the solubility difference of β-CyD and HP-β-CyD; HP-β-CyD has much higher water solubility than the unsubstituted β-CyD. 

The Uyar research group also reported hydroxypropyl cellulose (HPC) nanofibers impregnated with sulfisoxazole/CyD ICs [92]. Sulfisoxazole (SFS) is a sulfonamide drug with antibiotic properties [93]. Because of its low water solubility (i.e., <0.1 g per 100 mL), it was mixed with HP-β-CyD to form inclusion-complexation, which significantly increased the water solubility of SFS for enhanced therapy results. Thereafter, ICs were mixed with HPC, and the resultant mixture was electrospun to obtain ultrafine nanofibers. The IC-loaded nanofibers had a mean fiber diameter of 60 ± 25 nm, while sole SFS-loaded nanofibers (i.e., produced in the absence of CyD) sized 90 ± 40 nm, suggesting the use of ICs as an additive reduced the fiber diameter. However, the incorporation of IC did not make any significant impact on the fiber morphology. Although the initial release of SFS from the fiber matrix was fast, the complete release process took 12 h. The inclusion-complexation increased the release rate of SFS than those released from the HPC fiber matrix in the absence of CyD. Further, a sandwich-like fiber system was engineered by placing HPC/SFS/HP-β-CyD-IC nanofibers between PCL nanowebs. This nanofiber system slowed the release rate of SFS than the fiber prepared without PCL nanofibers. Tonglairoum et al. reported clotrimazole (CZ) loaded nanofibers through electrospinning [94]. A multicomponent fiber system containing CZ-loaded polyvinylpyrrolidone (PVP)/hydroxypropyl-β-cyclodextrin (HP-β-CyD) was prepared, and the fiber surface was coated with chitosan-cysteine (CS-SH)/poly(vinyl alcohol) (PVA) to increase the mucoadhesive properties and achieve the sustained release of CZ from the nanofibers. CZ-loaded nanofibers were successful in killing *Candida* significantly faster than the commercially used CZ lozenges at 5, 15, and 30 min and were safe for 2 h incubation. The release profiles of the embedded CZ did not show significant variations, and 80–90% release was observed after 480 min. Overall, the nanofibers showed promising results in the treatment of oral candidiasis. 

Voriconazole (VRC) is a triazole antifungal agent loaded as ICs with HP-β-CyD into poly(vinyl alcohol) (PVA) nanofibers and used for ophthalmic delivery [95]. The VRC-loading capacity could be enhanced with increasing CyD content. The total release of VRC took about 8 h from the nanofibers. Ocular irritation tests were performed on rabbit samples, and the Draize eye test results revealed that there was no ocular discomfort after the administration of the VRC nanofibers and VRC solution, suggesting that VRC-loaded PVA/HP-β-CyD composite nanofibers are promising non-irritant materials. Siafaka et al. reported voriconazole-loaded PCL nanofibers in the presence of β-CyD [96]. The ICs of VRC and β-CyD were mixed with PCL solution and electrospun to form nanofibers. VRC with various concentrations (5–20 wt.%) were loaded into nanofibers. All nanofibers showed an initial burst release of the VRC over 80%. The burst release was decreased with increasing VRC content. The nanofibers showed antifungal properties with the inhibition of *Candida albicans*. Opanasopit and co-workers reported fast releasing polyvinylpyrrolidone (PVP)/CyD composite nanofibers for the taste-masked meloxicam [97]. Meloxicam (MX) is a nonsteroidal anti-inflammatory drug molecule that is intended to be used in treating rheumatoid arthritis [98]. CyD molecules were blended to improve the fiber stability. MX was mixed with the CyD, and then PVP solution, of which the electrospinning led to nanofibers. In another CyD-based drug delivery system, electrospun mats were developed using PVP and HP-β-CyD/MX ICs [99]. MX was mixed with CyD molecules and electrospun in the presence of PVP in different solvent systems (dimethylformamide (DMF) and ethyl alcohol (EtOH)). HP-β-CyD molecules significantly improved the solubility of MX. A fast disintegration time and the burst release of MX were observed in an EtOH-based system. Furthermore, EtOH-based fiber system rapidly dissolved in the mouth. Cytotoxicity tests showed that the EtOH-system was much safer than the DMF-based system. Overall, the study showed a composite fiber of PVP and HP-β-CyD is ideal for fast-dissolving drug delivery to increase palatability of dosage forms. 

Recently, Monteiro et al. reported tetracycline/β-CyD IC loaded PCL nanofibers [100]. Tetracycline (TCN) is a common drug used for periodontal disease treatment [101]. After the preparation of TCN/β-CyD ICs, they were incorporated in PCL solution and electrospun to form nanofibers. The half of the embedded-TCN released as a burst from the matrix and then a slow release profile was observed. Total release of the entrapped TCN took 350 min. The nanofibers showed antimicrobial activity against *Aggregatibacter actinomycetemcomitans* and *Porphyromonas gingivalis*. TCN was also released in a sustained manner from CyD-free electrospun fibers of PCL/Zein [102,103] and PCL/poly(ethylene-*co*-vinyl acetate) (PEVA) [104,105]. The incorporation of PCL into the zein fibers slowed the release rate of TCN. TCN release could also be tuned by multi-layered electrospun fibrous mats. In this regard, Alhusein et al. showed that the sustained release of TCN from triple-layered fibers of PCL and PEVA when TCN loaded PEVA fibers were used in the middle covered by TCN-free PCL fibers from both sides [105]. This dramatically reduced the burst release of TCN, demonstrating the importance of drug localization on the release profile. In this context, a detailed review on the controlled release of drugs, including TCN, from PCL-based fibers was reported by Blagbrough and colleagues [106]. Chen and coworkers used silk-fibroin based electrospun nanofibers impregnated with HP-β-CyD as the carrier of Tamoxifen [107]. Tamoxifen (TAM) is a selective estrogen receptor modulator and acts as antiestrogen by binding to the estrogen receptors to inhibit the growth of the malignant mammary tumors in some tissues [108]. It is also effective in patients with ER-positive metastatic breast cancer [109]. TAM was mixed with HP-β-CyD molecules to form ICs to make it water-soluble. Thereafter, the solution was mixed with silk-fibroin and electrospun to form composite fibers. The inclusion-complexation significantly decreased the fiber diameter. The effective loading of TAM was confirmed by the Raman analysis of the fibers. The release results showed that the TAM first dissociated from the ICs, and then, was subsequently released from the fiber matrix. The release occurs slowly, and 50% release was observed after two weeks of incubation, suggesting that the presented concept can be intended as a promising local drug delivery system for breast cancer therapy. Souza et al. recently reported PCL nanofibers impregnated with β-CyD-silver sulfadiazine ICs [110]. Silver sulfadiazine (SAg) pertains to a sulfanilamide class of chemicals and is known for its topical antimicrobial properties, and is commonly used for the treatment of infected burns [111]. After inclusion-complexation with β-CyD, the solution was electrospun in acetic acid to form fibers, whose diameters showed great variations depending on the components; the solution of 15 wt.% PCL led to nanofibers with a mean diameter 358 nm, while IC-loaded PCL nanofibers possessed a mean diameter of 892 nm, suggesting the IC incorporation led to a dramatic increase in the fiber diameter. The release studies showed that, after one day, ca. 80% of SAg was released from CyD-free PCL fibers, while the respective value was 66% from the IC-loaded PCL fibers. The nanofibers demonstrated antibacterial properties against *S. aureus* and *E. coli*. 

Core-shell electrospun nanofibers impregnated with CyD functionalities have also been investigated in drug delivery applications. Generally, co-axial nanofibers promoted the sustained release of the entrapped drugs than the uniaxial fibers. In this regard, the Uyar research group reported the preparation of the core-shell nanofibers of curcumin/CyD IC and polylactic acid (PLA) [112]. The core of the nanofibers was IC of CyD and curcumin (CUR), while the shell layer was PLA. The formation of core-shell fiber structure was confirmed by TEM analysis with a contrast difference on the fibers. The slow release of CUR was achieved by core-shell nanofiber structure and the inclusion-complexation of CUR with HP-β-CyD provided high solubility. The embedded CUR showed antioxidant activity on the scavenging of 2,2-diphenyl-1-picrylhydrazyl (DPPH) radicals. CUR was also loaded into poly(vinyl alcohol) (PVA) nanofibers in a form of their complexes with CyD molecules [113]. The in vitro studies showed rapid diffusion of curcumin in the first hour followed by slower release. The nanofibers with low CUR loads showed faster release profiles. Another curcumin-loaded nanofiber system with multifunctional additives was developed using poly (l-lactic acid-*co*-ε-caprolactone) (PLACL) and Aloe Vera (AV), MgO nanoparticles, curcumin and β-CyD as a candidate material for breast cancer therapy [114]. These multifunctional nanofibers were tested with Michigan Cancer Foundation-7 (MCF-7) breast cancer cells. Depending on the fiber constituents, the proliferation of MCF-7 cells showed a clear decrease by 66% in PLACL/AV/MgO/CUR with respect to PLACL/AV/MgO nanofibrous scaffolds on day 9. The results demonstrated that 1% CUR interacting with MgO nanoparticles exhibited higher inhibition of MCF-7 cells among all other nanofibrous scaffolds, demonstrating their effectiveness for breast cancer therapy. Another PLLA- based nanofibers with CyD functionalities were prepared for the release of hydroxycamptothecin (HCPT) [115]. HCPT is an antitumor drug with poor water solubility, which restricts its use in clinical applications. The solution of PLLA and PEG was mixed with HP-β-CyD and HCPT and electrospun into nanofibers whose diameters slightly decreased from 780 to 700 nm in contrast to those nanofibers prepared in the absence of CyD and HCPT. The homogenous distribution of HCPT in the fiber matrix was confirmed by confocal analysis. The release studies revealed the initial burst release of ca. 15%, followed by a gradual release over 20 days. Increasing the HCPT content boosted the release percentage while the addition of higher CyD content minimized the burst release of HCPT. Recently, Masoumi et al. reported ciprofloxacin/CyD-encapsulated PCL nanofibers [116]. Ciprofloxacin (CIP) is an antibiotic drug molecule with poor water solubility and low dissolution rate, which limit its bioavailability [117]. CIP was mixed with α-CyD or β-CyD to form ICs and thereafter added to PCL solution. The electrospinning of these composite mixtures led to bead-free electrospun nanofibers loaded with CIP. The release tests were performed at pH = 7.2, and the released CIP content showed variations depending on the CyD-type and the preparation route of ICs; sonication-driven IC-loaded PCL fiber showed better release rate than those prepared without sonication. This can be ascribed to a higher amount of CIP entrapped in the CyD cavity under sonic energy. 

The ICs of antifungal drug griseofulvin (GSV) [118], antipsychotic drug (aripiprazole) [119] and phytochemical (asiaticoside) [120] with CyD were also mixed with various polymeric matrices and electrospun to form drug-loaded nanofibers. The Uyar research group reported allyl isothiocyanate loaded PVA nanofibers and investigated their antibacterial properties [121]. The ICs of antibacterial agent allyl isocyanate (AITC) and β-CyD were loaded into the electrospun nanofibers of PVA. CyD molecules significantly stabilized AITC during the electrospinning because of inclusion-complexation. The release experiments were performed at three different temperatures (30, 50 and 75 °C) in gas phase, and the release was dramatically increased with higher temperature. The nanofibers showed antibacterial properties with the significant inhibition of *E. coli* and *S. aureus*. AITC/CyD ICs were also embedded in the electrospun nanofibers of soy protein isolate (SPI), PEO or PLLA [122]. Fiber morphologies were affected by the AITC concentration used. The AITC release was negligible under dry conditions but increased dramatically as relative humidity rose. Luo et al. reported CyD functional polymeric nanofibers for sequential release of vascular disrupting and chemotherapeutic agents from electrospun nanofibers [123]. Combretastatin A-4 (CA4) and hydroxycamptothecin (HCPT) were loaded in the nanofibers of poly(ethylene glycol)-polylactide (PELA) in the presence of HP-β-CyD. The sequential release of CA4 and HCPT could achieve a sequential killing of endothelial and tumor cells. Anthelmintic drugs were also released from CyD functional electrospun fibers. In this context, Vigh et al. reported flubendazole/HP-β-CyD IC embedded PVP nanofiber webs with enhanced oral bioavailability [124]. A forty milligram could be released in 15 min, and the administration of the nanofiber system led to an increased plasma concentration profile in rats in contrast to the practically non-absorbable crystalline flubendazole. This can be ascribed to the effect of the embedded CyD, which prevented the crystallization of flubendazole. Doxorubicin (DOX), a chemotherapy medicine, was released from the PLLA nanofibers in the form of their ICs with methylated-β-CyD in parallel to their release from CyD-free PLLA fibers [125]. The results showed that the use of CyD molecules led to controlled release, and 17% decrease in the burst release was observed, followed by a quantifiable sustained release up to two days. Metoclopramide hydrochloride (MH), a drug molecule that has been used to treat nausea and vomiting, was incorporated into HP-β-CyD functional PVA nanofibers, and the release of MA was performed in a phosphate buffer (pH = 6.8) at 37 °C [126]. 90% Release of MA took place in 1 min due to burst release of MA. CyD-functional electrospun nanofibers were also used for the delivery of proteins. In one example, β-lactamase BlaP protein was mixed with the solution of chitosan/PEO containing CyD molecules, and the resultant mixture was electrospun into fibers [127]. CyD were used to increase the protein stability. In the absence of protein, spindle-shaped fibers were formed. However, the protein addition gave rise to smooth, bead-free nanofibers. The activity of the β-lactamase tested over the hydrolysis of nitrocefin, and enhanced hydrolysis was observed for the CyD functional nanofibers, demonstrating that the embedded CyD stabilized the protein in the fiber matrix. 

**Figure 4:**
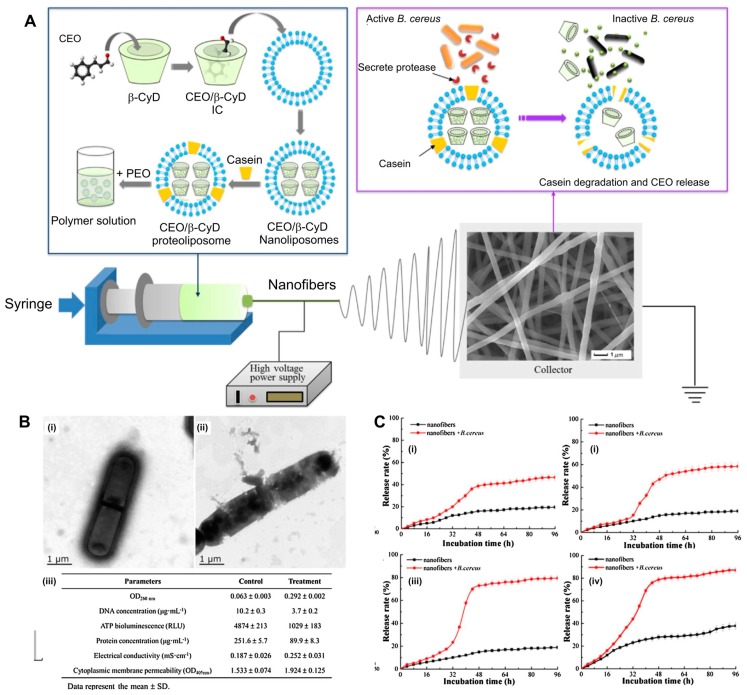
(**A**) Cartoon schemes of the production of CEO/β-CyD proteoliposomes incorporated poly(ethylene oxide) (PEO) fibers and *B. cereus* proteinase triggered cinnamon essential oil (CEO) delivery from CEO/β-CyD proteoliposomes. (**B**) TEM images of *B. cereus* before (**i**) and after the treatment (**ii**) of CEO/β-CyD proteoliposomes. (**iii**) The respective analysis results on the release of *B. cereus* cell constituents and the cell membrane permeability before and after proteoliposomes treatment. (**C**) The release rate of CEO/β-CyD proteoliposomes nanofibers stored at different temperatures 4 °C (**i**), 12 °C (**ii**), 25 °C (**iii**), and 37 °C (**iv**) for 4 days. The figure was reproduced from [133] with the permission of Elsevier, 2017.

A blending method was also applied for the preparation of essential oil loaded CyD functional electrospun fibers. Essential oils are plant-derived phytochemicals with antibacterial and antimicrobial properties [128,129]. They have also shown antimutagenic and antifungal activities [130]. Opanasopit and colleagues reported plai oil/HP-β-CyD IC [131] and herbal oil/ HP-β-CyD IC [132] loaded PVP nanofibers and monitored release profiles of the oil from the fiber matrix. The release of plai oil from the fiber matrix demonstrated burst release and subsequent sustained release over 24 h. The release rate changed between 10 and 30% in 24 h depending upon the oil load in the fiber. The release of herbal oil took less than one minute. Lin et al. reported cinnamon essential oil (CEO)/CyD loaded PEO nanofibers [133]. In this regard, first CEO/β-CyD proteoliposomes were prepared by using a thin-lipid film evaporation-ultrasonic hydration-freeze and thaw technique. Afterwards, they mixed with PEO and electrospun to form bead-free nanofibers (Figure 4A). The CEO release studies were performed in ethanol, and the amount of CEO released from the fiber matrix was measured by GC-MS. In the presence of *Bacillus cereus*, the release of CEO was significantly enhanced. The CEO release from the nanofibers was about 30% in 96 h, whereas this increased to 80% in the presence of *B. cereus*. A similar effect was observed at higher temperature, which enhanced the release rate of CEO (Figure 4C). The resultant nanofibers showed significant inhibition against the growth of *B. cereus* due to the antibacterial property of CEO. 

The cinnamon essential oil (CEO) was also encapsulated in PVA/β-CyD nanofibers, and in this context, the ICs of cinnamon and β-CyD were mixed with PVA solutions and electrospun to form nanofibers with a mean diameter of 240 ± 40 nm [134]. CyD molecules notably increased the thermal stability of CEO after complexation. Due to the intrinsic antimicrobial property of CEO, the nanofibers exhibited strong antimicrobial activity against *E. coli* and *S. aureus*. Furthermore, the entrapment in fiber systems prolonged the shelf-life of CEO. Cinnamon/β-CyD ICs were also loaded into PLLA fibers, and the nanofibers showed antimicrobial properties [135]. The same research group also reported an identical fiber system using lysozyme as an additive and observed the enhancement of the antimicrobial activity of cinnamon essential oil-loaded electrospun nanofilm by the incorporation of lysozyme [136]. Recently, Munhuweyi et al. reported that cinnamon and oregano essential oils–loaded PVA-CyD nanofibers [137]. The ICs of essential oils with β-CyD molecules were prepared and mixed PVA solutions, of which the electrospinning led to nanofibers varying between 120 and 180 nm in diameter depending on essential oil loading. Liu et al. reported cinnamaldehyde/CyD loaded PLLA fibers via electrospinning [138]. Cinnamaldehyde (CA) pertains to the class of essential oils and is known for its antibacterial and antioxidant properties [139]. The ICs of the β-CyD and CA were mixed with PLA and electrospun into fibers of 4–6 μm depending on the IC loading. Increasing IC content in the PLLA fiber decreased water contact angle because of many OH groups. Increasing IC content led to high release percent of the embedded CA. In the first 20 h, a significant increase of CA was observed, but afterwards, almost no change on the release profile was monitored. The CA-loaded fibers showed antibacterial properties against *E. coli* and *S. aureus.* Likewise, ultrafine nanofibers from zein impregnated with the ICs of eucalyptus essential oil (EEO) and β-CyD were produced by electrospinning [140]. The ICs of EEO and β-CyD were prepared by co-precipitation technique and added to aqueous ethanol solutions of zein. The electrospinning of the mixtures led to the formation of bead-free nanofibers. The IC-loaded zein nanofibers showed 24% greater reduction of growth than pure zein fibers. For *Listeria monocytogenes,* the growth reduction was 28.5%, and for *Staphylococcus aureus,* it was 24.3%. 

PLLA was also used as a carrier of gallic acid (GA)/CyD ICs [141]. The IC of GA with HP-β-CyD molecules was prepared and mixed with PLLA solutions whose electrospinning led to bead-free nanofibers. The release studies were performed in aqueous solution of EtOH and increased GA release was observed with increasing EtOH content. The release came to equilibrium after 4 h. The nanofibers showed antioxidant activity in the scavenging of DPPH radicals. Mascheroni et al. reported perillaldehyde-loaded pullulan nanofibers functionalized with β-CyD [142]. The blending of pullulan and β-CyD with embedded perillaldehyde was performed and electrospun. The release of volatile perillaldehyde showed a profound effect of RH on the release profile. 

The Uyar research group has reported several studies related to the encapsulation of CyD ICs of volatile active agents such as essential oils (e.g., eugenol [85], geraniol [86]) or fragrance/flavor molecules (e.g., menthol [143,144,145], vanillin [146]) into electrospun polymeric nanofibers in order to enhance the temperature stability and shelf-life of such volatile active agents. Likewise, hexanal, a naturally occurring volatile compound, released from CyD-functional xanthan fibers. The CyD-functional xanthan fibers showed faster release of hexanal than pure xanthan fibers [147]. Moreover, a type of vitamin E (α-tocopherol), an antioxidant, was incorporated into PLLA nanofibers after its complexation with γ-CyD molecules [148]. The PLLA/α-tocopherol/γ-CyD nanofibers showed antioxidant properties. The Uyar research group has also reported β-CyD/quercetin ICs loaded poly(acrylic acid) (PAA) nanofibers [149]. The electrospinning of the mixture of PAA, β-CyD and quercetin led to well-tuned nanofibers with a mean diameter of 270 nm. The nanofibers showed a burst release of almost all quercetin molecules. The nanofibers showed antioxidant activity due to the embedded quercetin. Kingshott and colleagues reported retinyl acetate (RA) release from β-CyD functional PVA nanofibers [150]. The ICs of β-CyD and RA were incorporated into PVA nanofibers to enhance the shelf-life and thermal stability of RA. The release of RA was very slow, reaching 90% after 3 months. The general overview of the CyD/drug ICs loaded polymeric electrospun nanofibers was summarized in Table 1, where the composition of the fiber matrix and drug molecules were given, along with release data. 

### 4.2. Poly-Cyclodextrin Functional Electrospun Nanofibers for Drug Delivery Systems

**Figure 5:**
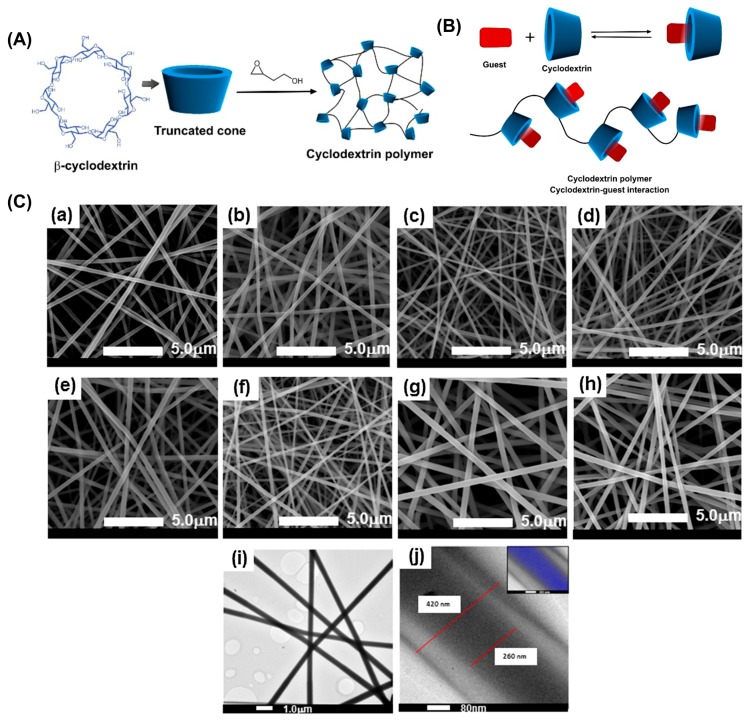
Cartoon schemes of (**A**) the synthesis pathway of CyD polymers and (**B**) their complexation with guest molecules. (**C**) scanning electron microscopy (SEM) and transmission electron microscopy (TEM) images of uni- and co-axial PMAA/polyCyD fibers: (**a**) SEM uniaxial–PMAA; (**b**) SEM uniaxial–PMAA + PROP; (**c**) SEM uniaxial PMAA/polyCyD (80:20); (**d**) SEM uniaxial PMAA/polyCyD (80:20) + PROP; (**e**) SEM uniaxial PMAA/polyCyD (60:40); (**f**) SEM uniaxial PMAA/polyCyD (60:40) + PROP; (**g**) SEM coaxial–shell (PMAA) and core (polyCyD); (**h**) SEM coaxial–shell (PMAA) and core (polyCyD + PROP); (**i**) TEM coaxial–shell (PMAA) and core (polyCyD + PROP); and (**j**) TEM coaxial–shell (PMAA) and core (polyCyD + PROP). The figure was reproduced from [151] with the permission of Elsevier, 2015.

CyD can be cross-linked or polymerized in controlled or noncontrolled routes to form cyclodextrin based polymeric materials (poly-cyclodextrin (polyCyD)). Further, CyD can be functionalized with polymerizable groups to produce controlled CyD polymers. The poly-cyclodextrin (polyCyD) was also electrospun to form nanofibers to be used in drug delivery applications. In this regard, Oliveira et al. reported coaxial nanofibers based on polyCyD associated with poly(methacrylic acid) (PMAA) for the release of hydrophilic drug, propranolol hydrochloride (PROP) [151]. The nanofibers were cross-linked with thermal treatment at 170 °C for 48 h to obtain water-insoluble polyCyD nanofibers (Figure 5a–c). The biocompatibility of the nanofibers was explored over fibroblast cells, and high cell viability was observed. The interaction between CyD and drug molecules was explored and found to be spontaneous. The burst release of the encapsulated PROP could be modulated by coaxial electrospinning. Uniaxial nanofibers produced with PMAA/polyCyD (80:20 *w*/*w*) and (60:40 *w*/*w*) released 30% and 35% PROP in 8 h, while sole PMAA nanofibers released 100% dose in 15 min. Further, coaxial nanofibers made by polyCyD/PROP core and a shell of PMAA showed better sustained release. In another study, polyCyD was used for the release of fluconazole from the uniaxial nanofibers of polyCyD mixed with PCL and PVP [152]. The nanofibers were further coated with a hydrophobic poly(hexamethyldisiloxane) (polyHMDSO) for tailoring the fiber dissolution and the respective drug release rate. The coating process was performed under mild condition plasma polymerization. Unlike non-coated fibers, which rapidly released the drug payload, polyHMDSO-coated nanofibers prolonged drug releasing time to 24 h. The coated nanofibers were tested with bacteria, and the results showed that they could inhibit the in vitro growth of *Candida albicans*. Another polyCyD-based electrospun nanofiber system was developed using a polymer of HP-β-CyD/citric acid mixed with chitosan to obtain polyelectrolyte complexes [153]. Triclosan was mixed with polyHP-β-CyD to form ICs, and thereafter, chitosan was added to the solution. The nanofibers were cross-linked with a treatment at 90 °C for 4 h and demonstrated antimicrobial activity against the growth of *S. aureus* and *E. coli* for longer periods of time. 

Hydrophobic CyD-based polymers were also used for drug delivery applications. In this regard, Heydari et al. reported the controlled release of vitamin B2 from hydrophobic peracetyl-β-CyD polymer (Acβ-CyDP) based electrospun nanofibers [154]. Acβ-CyDP was synthesized by the acetylation of epichlorohydrin functional β-CyD polymer in the mixture of acetic acid and pyridine at 100 °C for 7 h. The electrospinning of Acβ-CyDP in the mixture of acetone and DMF (3:2, *v*/*v*) led to thin nanofibers, of which the diameters remained nearly stable even with increasing the polymer concentration from 8 to 25 wt.%, demonstrating no clear influence of CyD content on the diameters of the resultant fibers. The cumulative release of vitamin B2 from the fiber matrix was explored at two different pH values (1.2 and 7.4), and the results revealed enhanced release at pH = 1.2. The release of vitamin B2 gradually occurred, and 60 and 40% of the vitamin diffused out of the nanofibers after 170 h for the solutions having pH of 1.2 and 7.4, respectively. Nada et al. used a CyD cross-linked gelatin fiber matrix for the carrier of chloramphenicol [155]. Chloramphenicol is an antibiotic, which may lead to bone marrow suppression and influences red blood cells [156]. The oxidation of β-CyD led to polyaldehyde β-CyD, and it was used to cross-link gelatin. The nanofibers showed antimicrobial activities against *Staphylococcus aureus* (Gram positive) and *Pseudomonas aeruginosa* (Gram negative bacteria) and *Candida albicans*. After two days, the release was as ca. 90%. A water-insoluble anti-inflammatory drug atorvastatin was loaded in PCL fibers after its complexation with poly-amino-CyD molecules [157]. The quantity embedded was estimated (70–90 μg in 30 μm × 6 mm membrane) and the anti-inflammatory effect by cell contact-dependent release reached 60% inhibition for TNF-α and 80% for IL-6. Drug delivery electrospun nanofibers were also developed using PVA and carboxymethyl-β-CyD grafted chitosan [158]. The carboxymethyl-β-CyD was grafted on chitosan by 1-ethyl-3-(3-dimethylaminopropyl) carbodiimide (EDC) and *N*-hydroxysuccinimide (NHS) based coupling. Nanofibers between 130 and 210 nm in diameter were obtained. The presence of CyD molecules in the nanofibers slowed the release rate of salicylic acid (SA). After an initial burst release of SA, the release percentage reached over 80% after 24 h. CyD-functional fibers were also produced by the electrospinning of a CyD functional polymer and an antibiotic drug vancomycin [159]. The CyD-functional polymer was synthesized by poly(ethylene-vinyl alcohol) (pEVOH)/thiol-modified CyD and a bifunctional cross-linker *N*-(p-maleimidophenyl) isocyanate (PMPI). The fibers showed the sustained release of vancomycin. CyD-functional polymeric nanofibers were also used to the delivery of a common insect repellent, *N*,*N*-diethyl-3-toluamide (DEET) [160]. Micro-sized fibers with a diameter of 2.8 ± 0.8 μm were obtained by the electrospinning of a water-soluble β-CyD/pyromellitic dianhydride (PMDA) polymer and thereafter loaded with DEET in diethyl ether. The release data were obtained by TGA analysis, which revealed a sustained release of all DEET in two weeks. The details related to the polyCyD/drug nanofibers for drug delivery were summarized in Table 2. 

### 4.3. Polymer-Free Cyclodextrin Electrospun Nanofibers for Drug Delivery Systems

Drug-loaded electrospun CyD nanofibers can also be prepared without using a polymeric carrier matrix. The electrospinning of polymer-free CyD/drug IC solutions can produce uniform nanofibers, which are solely based on CyD molecules and their ICs with drug molecules. As these electrospun nanofibers are mostly uncross-linked and hydrophilic, rapid release profiles were observed, along with the fast-dissolving fiber character. The electrospinning of CyD molecules takes place over their aggregates, which are governed by numerous hydrogen bonds. The Uyar research group has pioneered polymer-free electrospinning of nanofibers from CyD and CyD-IC based systems. The first paper was reported in 2010 [161], and it has been shown that highly concentrated (140–160 wt.%) solutions of methylated β-CyD (M-β-CyD) molecules either in water or dimethylformamide (DMF) form large aggregates driven by hydrogen bonds, which is the key factor for uniform fiber formation during electrospinning process [161]. In a later study, polymer-free nanofibers were successfully electrospun from three different CyD derivatives—HP-β-CyD, HP-γ-CyD and M-β-CyD—in three different solvent systems—water, DMF, and dimethylacetamide (DMAc) [162]. The polymer-free nanofibers from native CyD were also produced, in which electrospinning was carried out for α-CyD and β-CyD in alkaline aqueous systems [83], and γ-CyD nanofibers were electrospun from a dimethyl sulfoxide (DMSO)/water solvent system [163]. The Uyar research group has also shown that the electrospinning of uniform nanofibers could be produced from CyD-ICs without using a polymer carrier, in which triclosan/CyD ICs system was successfully electrospun into uniform and bead-free nanofibers [164]. In another study, antibacterial properties of electrospun nanofibers from triclosan/CyD IC using two different CyD derivatives (HP-β-CyD and HP-γ-CyD) were also reported [165]. Triclosan/CyD ICs electrospun nanofibers showed better antibacterial properties in the inhibition of growth of Gram-negative (*E. coli*) and Gram-positive (*S. aureus*) bacteria than pure triclosan. 

Essential oils are widely implemented in medical, food, cosmetic, and allied applications due to their antibacterial, antiseptic, antifungal, and antioxidant properties [139]. The Uyar research group has made significant contributions in the fabrication of essential oil loaded CyD functional nanofibers, and several studies related to the polymer-free electrospinning of essential oils/CyD ICs were reported. Since the most of them are slightly water soluble, the use of CyD molecules in the nanofiber form can offer bulk materials loaded essential oils. Very recently, Celebioglu et al. has reported the electrospinning of CyD-camphor IC nanofibers [166]. Camphor-loaded CyD nanofibers rapidly dissolved in water because of their uncross-linked hydrophilic nature. Despite the fact that camphor is a volatile molecule, it can be preserved in the CyD cavity during electrospinning, and the molar ratio after electrospinning was calculated as ~1.00/0.65 and ~1.00/0.90 in HP-β-CyD/camphor-IC and HP-β-CyD/camphor-IC nanofibers, respectively. The camphor release from HP-β-CyD-IC and HP-γ-CyD-IC nanofibers in gas phase was monitored at two different temperatures (37 and 75 °C) using gas chromatography-mass spectrometry (GC-MS) for 4 h, where the higher temperature enhanced the release rate of camphor because of the diffusion coefficient increment of camphor molecules. Another important essential oil, eugenol, was complexed within CyD nanofibers [167]. The ICs of three cyclodextrin derivatives (HP-β-CyD, HP-γ-CyD, and M-β-CyD) with eugenol were prepared in water and electrospun to form nanofibers. Inclusion-complexation significantly enhanced water solubility and increased the thermal stability of volatile eugenol. Due to their hydrophilic and uncross-linked nature, CyD nanofibers rapidly dissolve in water. The eugenol-loaded CyD nanofibers showed a higher antioxidant capacity than eugenol itself, suggesting enhanced antioxidant activity of the fibers than the powder form of eugenol. Essential oils, *p*-cymene and cineole, were also embedded in polymer-free CyD nanofibers in the form of CyD-ICs [168]. Electrospun nanowebs were produced by the ICs of *p*-cymene and cineole with two modified cyclodextrins (HP-β-CyD and HP-γ-CyD). The thermal stability of both *p*-cymene and cineole increased with inclusion-complexation. On contact, the nanofibers rapidly dissolved in water. Linalool, a natural ingredient of many essential oils, was also loaded into CyD nanofibers as ICs through electrospinning [169]. Well-tuned nanofibers were obtained with antibacterial properties. Antibacterial limonene-loaded polymer-free CyD nanofibers were prepared by electrospinning [170]. Modified cyclodextrins (M-β-CyD, HP-β-CyD and HP-γ-CyD) and limonene were mixed to form ICs. The computational and experimental results revealed the molar ratio of the CyD-limonene was 1:1. The released limonene from M-β-CyD/limonene-IC was less than the nanofibers of HP-β-CyD/limonene-IC and HP-γ-CyD/limonene-IC due to the higher amount of the preserved limonene in the nanofiber. 25% limonene was released from M-β-CyD/limonene-IC nanofibers, whereas the nanofibers of HP-β-CyD/limonene-IC and HP-γ-CyD/limonene-IC released 51 and 88 wt.% limonene in 100 days, respectively. Inclusion-complexation significantly enhanced the thermal stability of the entrapped active molecules. The nanofibers significantly inhibited the growth of *S. aureus* and *E. coli* because of the antibacterial property of limonene. In addition, the nanofibers rapidly dissolved in water because of the polymer-free nature of hydrophilic CyD derivatives. Geraniol-CyD ICs-loaded nanofibers were also reported by the Uyar research group without the requirement of any polymer carrier [171]. Bead-free nanofibers were obtained using three different CyD types e.g., HP-β-CyD, M-β-CyD, and HP-γ-CyD. Even though geraniol is a volatile molecule, it could be preserved in the nanofibers in the range of ∼60–90% depending on formulation parameters. The release studies were either performed at room temperature or short term at high temperature (37, 50, and 75 °C), and the results revealed M-β-CyD /geraniol-IC complex loaded nanofibers released less geraniol than the nanofibers of HP-β-CyD/geraniol-IC and HP-γ-CyD/geraniol-IC, suggesting the strongest inclusion-complexation occurred between M-β-CyD and geraniol. The nanofibers showed antibacterial properties in the sense of the inhibition of growth of *E. coli* and *S. aureus* because of the released geraniol from the fiber matrix. Furthermore, the nanofibers showed enhanced antioxidant activity to those of pure geraniol because of its low water solubility. Likewise, thymol-loaded CyD nanofibers were prepared as ICs of CyD (M-β-CyD, HP-β-CyD, and HP-γ-CyD) [172]. Due to the volatile nature of thymol, it was entrapped in the CyD cavity to enhance its thermal stability. Thymol-loaded CyD nanofibers showed more rapid dissolution in water than hydrophobic thymol. Due to the antioxidant properties of thymol, the respective nanofibers were tested by DPPH radical scavenging, and high scavenging capacity was observed. Polymer-free CyD nanofibers were also electrospun from their ICs with volatile flavor agents e.g., menthol [173] and vanillin [174], and vitamins e.g., vitamin E [175] with enhanced solubility and prolonged shelf-life. Recently, Uyar and colleagues reported the polymer-free electrospinning of carvacrol/CyD ICs [176]. Carvacrol is a phenolic component of plant essential oils and has antioxidant and antimicrobial properties [177,178]. The ICs of carvacrol with HP-β-CyD or HP-β-CyD were electrospun into fibers. These fast dissolving fibers showed enhanced antioxidant activity with an increase content of carvacrol. 

**Figure 6:**
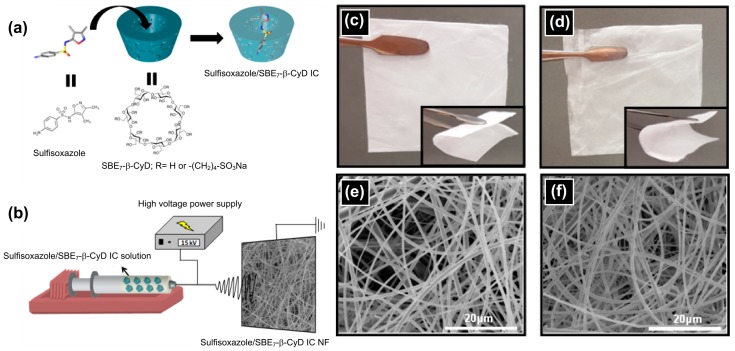
(**a**) Cartoon illustration of inclusion-complexation between CyD and sulfisoxazole (SFS). The chemical structure of sulfisoxazole and SBE_7_-β-CyD with a schematic representation of sulfisoxazole, SBE_7_-β-CyD and their IC, (**b**) schematic representation of the electrospinning of SFS/SBE_7_-β-CyD-IC NF. Photographs of electrospun (**c**) SBE_7_-β-CyD nanofibers, (**d**) SFS/SBE_7_-β-CyD-IC nanofibers, and SEM images of (**e**) SBE_7_-β-CyD NF, (**f**) SFS/SBE_7_-β-CyD-IC nanofibers. The figure was reproduced from [179] with the permission of Elsevier, 2017.

**Figure 7:**
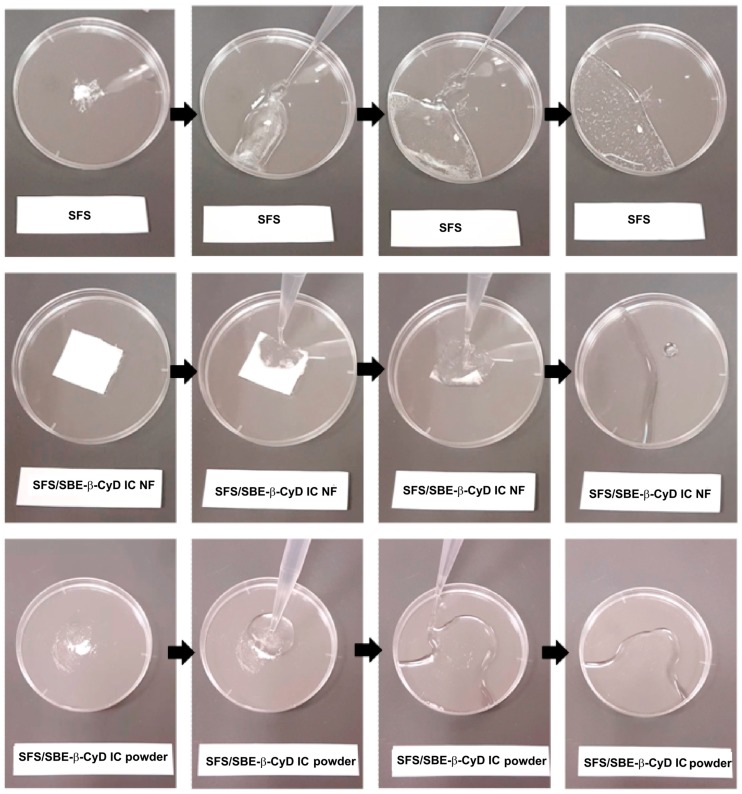
Typical water-solubility of the drug loaded polymer-free CyD fibers. The representative photos of the SFS and SFS/ SBE_7_-β-CyD IC powder and SFS/ SBE_7_-β-CyD IC nanofibers on exposure to water. The figure was reproduced from [179] with the permission of Elsevier, 2017.

In order to develop a fast-dissolving drug/CyD IC nanofibrous system, the ICs of sulfisoxazole (SFS) and sulfobutyl ether β-CyD (SBE_7_-β-CyD) were prepared in water, and thereafter, electrospun into nanofibers in the form of self-standing flexible nanofibrous webs (Figure 6) [179]. In parallel, the ICs of SBE_7_-β-CyD and SFS were prepared as powder. On contact with water, the entrapped SFS was released from the polymer nanofibers, along with the disintegration of the fibers (Figure 7). The polymer-free nanofibers of SBE_7_-β-CyD-SFS ICs showed faster dissolution than the powder form of the SBE7-β-CyD-SFS ICs, and pure SFS was not soluble in water in the absence of CyD (Figure 7). These fast dissolving CyD functional fiber nanowebs can be implemented if the rapid release of water-insoluble drugs, as the case here with SFS, is desired.

**Figure 8:**
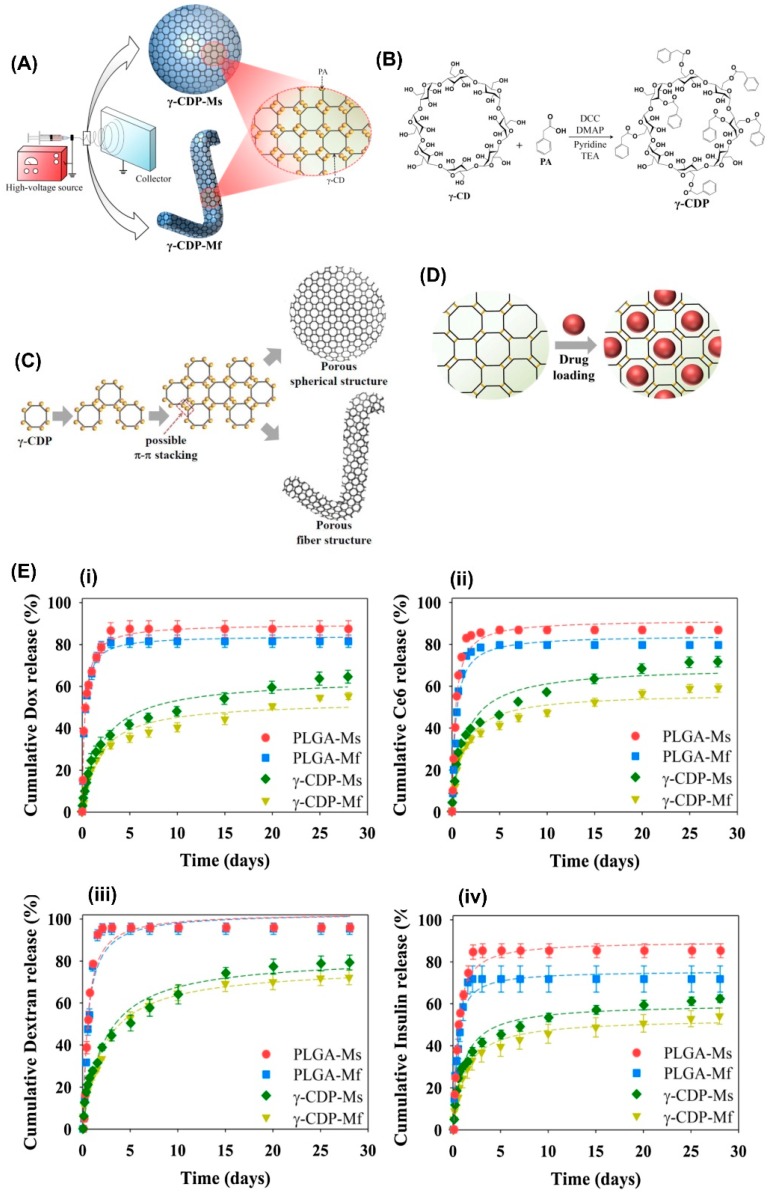
(**A**) A schematic representation of the electrospinning and electrospraying of γ-CyDPs. (**B**) The synthesis pathway of γ-CyDP. (**C**) Cartoon schemes of γ-CyDP-microspheres (Ms) or γ-CyDP microfibers (Mf) with porous structure and (**D**) their drug loading. (**E**) The cumulative molecule (**i**) Dox, (**ii**) Ce6, (**iii**) dextran, and (**iv**) insulin release (wt. %) from PLGA-Ms, PLGA-Mf, γ-CyDP-Ms, and γ-CyDP-Mf (*n* = 3). The figure was reproduced from [182] with the permission of Elsevier, 2018.

Another fast-dissolving polymer-free fiber system loaded with poorly soluble diclofenac sodium was reported by Balogh et al. [180]. The fibers rapidly dissolved in water (within 2 min). Spironolactone, a diuretic drug with antiandrogenic properties, was complexed with CyD and electrospun into nanofibers without the requirement of any polymeric carrier [181]. The resultant nanofibers rapidly dissolved in water, and the system allowed high loading of lipophilic spironolactone. An interesting CyD based polymer-free fiber system for drug delivery applications was developed using modified γ-CyD molecules. Yu et al. modified γ-CyD with phenylacetic acid and performed electrospinning from the mixed solution of dichloromethane (DCM) and dimethylformamide (DMF) (8:2, *v*/*v*) to form fibers (Figure 8) [182]. The bead-free, water insoluble ultrafine fibers with a diameter range of 1–2 μm were obtained. Unlike other polymer-free CyD fibers, these fibers did not show rapid dissolution. In vitro cell tests revealed the fibers are biocompatible. Several drug molecules (doxorubicin, fluorescein isothiocyanate-dextran (FITC-dextran), recombinant human insulin (FITC-labeled insulin), and chlorin) were loaded by simply incubating the fibers in the solutions of the respective drug molecules. ~50% of Release of the entrapped drug molecules was observed from the fiber matrix in one month, demonstrating their slow release (Figure 8E). Chlorin-loaded fibers were also exploited for in vivo drug release in mice, and on day 28, the complete release of chlorin was observed because of the disappearance of its fluorescence signal. The general overview of polymer-free CD/drug ICs fibers for drug delivery was summarized in Table 3. 

## 5. Concluding Remarks and Future Outlook

**Figure 9:**
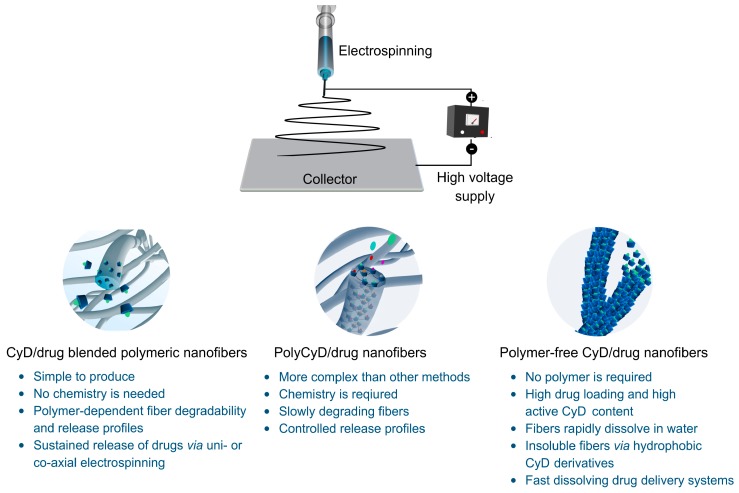
Cartoon illustration of CyD-functional electrospun nanofibers used for drug delivery applications.

Electrospinning has become a dynamic, rapidly changing field that can affect human lives in diverse ways. It allows for the production of electrospun nanofibers with tailored-made properties to address problems in the environment, biomedicine, textile, and food industries. High specific surface area, ease of operation, adaptability, tunable fiber texture, and a wide size spectrum make the electrospun nanofibers potential drug carriers with many intrinsic benefits, including enhanced drug loading, controlled release of drugs, and a short diffusion pathway. Further, processing parameters and structural modification allow tuning the fiber structure for the sustained release of drug molecules—for example, in the case of co-axial nanofibers with the drugs-embedded in the core. In this regard, various drug molecules with anticancer, antibiotic, antimicrobial, or antioxidant properties were incorporated into electrospun fibers, mostly using their ICs with CyD. The non-specific incorporation of such drug molecules can cause undesired release profiles, low drug loadings, activity loss of environmentally sensitive drugs, or other problems. In this regard, the incorporation of biocompatible nanocarriers into such systems allows the fabrication of high-performance drug delivery nanomaterials. Toward this goal, CyD molecules have found a large application area because of their unique 3D structure, which endows nanofibers with distinct features e.g., enhanced drug solubility, stability, and bioavailability useful in drug delivery applications. Furthermore, they do not provoke the immune system and have low toxicities to mammals. Hence, they have become important excipients in pharmaceutical applications. Native CyD, particularly β-CyD, have ideal cavity size related to complex with a wide range of chemicals but have significant issues related to the poor water solubility of native CyD. These drawbacks of β-CyD can be mitigated by chemical modifications to produce highly soluble derivatives, such as hydroxypropyl functional-CyD. CyD-functional electrospun nanofibers were prepared by either blending of CyD-drug complexes with polymers or using CyD polymers with another polymer carrier or standalone. CyD molecules could also be electrospun without the requirement of a carrier polymer. In the latter case, CyD and guest molecules were mixed to form ICs and their electrospinning produced polymer-free nanofibers with high drug loading. However, these fibers, on contact, rapidly dissolve in water and release the entrapped drug molecules in the form of CyD-drug ICs without any control on drug release. Table 1, Table 2 and Table 3 give the characteristics of some selected examples on CyD functional electrospun nanofibers used in drug delivery applications. All these fiber systems have their own pros and cons. Still, there is a growing interest to engineer CyD-functional drug delivery systems that deliver therapeutics in a safe, effective, and targeted fashion. The most of researches in this field concentrated on the blending of CyD-drug ICs with various polymers. This route produces CyD-drug IC loaded polymeric nanofibers with longer dissolution time depending on the structural properties of polymeric carrier in the fiber. The CyD-drug ICs can also be electrospun in the absence of a polymer. However, such fibers rapidly dissolve on contact with water, and drug molecules are released in the forms of CyD-drug ICs. In contrast to both methods, the polyCyD fibers can be engineered and used in drug delivery with a better control on release profiles. A schematic overview of the CyD-functional nanofibers was shown in Figure 9, where important parameters of the relevant synthesis routes are pointed out. Each preparation approach comprises different benefits. While polymer-free CyD nanofibers are simpler to produce, they suffer from the instant water solubility. However, in this context, hydrophobically-modified CyD can offer water-insoluble polymer-free fiber meshes. On the other hand, polyCyD functional nanofibers have certain difficulties in the synthesis of functional CyD derivatives and their polymers. Regardless of preparation route, the ability to tune fiber structure should facilitate the sustained release of drugs, such as through co-axial electrospinning. 

The use of CyD in electrospun nanofibers for drug delivery systems are likely to find increasing interest in the coming years. Currently, there are significant advances in polyCyD electrospun nanofibers produced without the need of any additional polymeric carrier. These water insoluble nanofibers exhibited the efficient removal of water micropollutants, e.g., methylene blue, because of their high active CyD contents [183]. In the future, such nanofibers may find applications in drug delivery. Further, a stimulus can be applied for the release of drug molecules from the CyD cavity in a controllable fashion. Likewise, CyD-containing polymers, particularly those contain CyD as pendent motifs on the polymer backbone can offer promising results for the sustained release of drugs from the fiber matrix depending on the hydrophobicity of the polymer. In this regard, cross-linked nano-porous β-CyD polymers showed enhanced IC constant as high as 10^9^ M^−1^ than those uncross-linked ones (native β-CyD, 10^3^ M^−1^) [184]. Beside their use in in vitro drug release applications, CyD-functional electrospun materials will find more applications as implant materials in tissue engineering. Unlike CyD-free electrospun fibers, the functionalization of electrospun fibers with CyD can improve their properties in terms of high drug loading, increased stability, and bioavailability of the payloads in the body. 

To conclude, drug delivery is a dynamic and complex process and can be adapted to meet the needs of the targeted application. Advanced drug delivery therapy requires nontoxic pharmaceutical excipients to provide high drug loading and keeps the drugs stable, active, and accessible for bioavailability. In this regard, CyD-functional nanofibers would be one of the best material systems for drug delivery applications since they offer intrinsic features of both electrospun nanofibers and CyD possessing a hydrophobic molecular-environment for drug entrapment. CyD, thus, significantly increase water solubility of hydrophobic drug molecules (i.e., for high drug loading) and shield drug molecules from physiological degradation or elimination for bioavailability while nanofiber form offers high specific surface area, ease of operation, and further control on the nanofiber form for the sustained release of the entrapped drug molecules. As the electrospinning produces tailored nanostructured materials with further control mechanisms on drug release, the utilization of CyD-functional electrospun materials will increase to meet the demands of advanced drug delivery systems.

## Figures and Tables

**Table 1:** General overview of CyD/drug ICs embedded polymeric electrospun nanofibers used in drug delivery.

CyD Type	Polymer Additive	Active Molecule	Release Data	Ref
β-CyD	PCL	Naproxen (NAP)	Higher NAP release with CyD	[88]
β-CyD, HP-β-CyD	Pellethane (TPU)	Naproxen (NAP)	10 h (NAP-TPU), 32 h (NAP/β-CyD/TPU), 120 h (NAP, HP-β-CyD/TPU)	[91]
HP-β-CyD	Hydroxypropyl cellulose (HPC)	Sulfisoxazole (SFS)	720 min (PCL-PCL-HPC/SFS/HP-β-CyD-IC-NF), >720 min (HPC/SFS/HP-β-CyD-IC-NF)	[92]
HP-β-CyD	PVP, PVA, Thiolated chitosan (CS-SH)	Clotrimazole (CZ)	For all nanofibers 80% in 480 min	[94]
HP-β-CyD	PVA	Voriconazole (VRC)	8 h for 100% release	[95]
β-CyD, HP-β-CyD	PVP	Meloxicam (MX)	For all nanofibers, 20 min for 100% release	[97]
HP-β-CyD	PVP	Meloxicam (MX)	Rapid release (<10 min)	[99]
β-CyD	PCL	Tetracycline (TCN)	Drug release occurred up to 2 weeks	[100]
HP-β-CyD	Silk fibroin (SF)	Tamoxifen (TAM)	10% in 22 days in PBS, 50–60% in PBS-EtOH (30%) in 22 days)	[107]
β-CyD	PCL	Silver sulfadiazine (SAg)	80% release from PCL/SAg, 66% release from PCL/SAg/β-CyD	[110]
HP-β-CyD	PLLA	Curcumin (CUR)	Higher release at pH of 1. CyD increased drug release.	[112]
β-CyD	PVA	Curcumin (CUR)	Higher drug content increased the release rate.	[113]
β-CyD	Poly (l-lactic acid-co-ε-caprolactone) (PLACL)	Curcumin (CUR)	1% CUR interacting with MgO nanoparticles showed higher inhibition of breast cancer cells.	[114]
HP-β-CyD	Poly(dl-lactic acid)–poly(ethylene glycol) (PELA)	Hydroxycamptothecin (HCPT)	Higher CyD content increased release rate. The release was slow and took many weeks.	[115]
α-CyD and β-CyD	PCL	Ciprofloxacin	Higher release with initial higher drug loading	[116]
SBE-β-CyD	PEO	Aripiprazole (ARP)	Rapid release in 2 min	[119]
HP-β-CyD	Cellulose acetate	Asiaticoside (AC)	Higher release with CyD and initial burst release within 300 min	[120]
β-CyD	PVA	Allyl isothiocyanate (AITC)	Higher release at 75 °C and followed by 50 and 30 °C.	[121]
β-CyD	PEO	Allyl isothiocyanate (AITC)	Higher release with increasing relative humidity	[122]
HP-β-CyD	Poly(ethylene glycol)-polylactide (PELA)	Combretastatin A-4 (CA4) and hydroxycamptothecin (HCPT)	Sustained release of CA4 over 30 days, fibers showed significant antitumor efficacy and tumor vasculature destruction	[123]
HP-β-CyD	PVP	Flubendazole	The release of a dose of 40 mg in 15 min	[124]
M-β-CyD	PLLA	Doxorubicin (DOX)	17% Decrease in the burst release was observed and followed by a quantifiable sustained release up to 2 days.	[125]
HP-β-CyD	PVA	Metoclopramide hydrochloride (MH)	Burst release: 90% release in 2 min	[126]
β-CyD derivative	Chitosan	β-Lactamase BlaP protein	CyD increased the stability of the embedded protein	[127]
HP-β-CyD	PVP	Plai oil	The release rate ranged was in the order of 10% > 20%~30% plai oil within 24 h.	[131]
HP-β-CyD	PVP	Herbal oil	Very rapid release: 50% release in 1 min	[132]
β-CyD	PEO	Cinnamon (CEO)	Controlled release in nanofibers via bacterial protease.	[133]
β-CyD	PVA	Cinnamon (CEO)	Nanofibers showed excellent antimicrobial activity against *E. coli* and *S. aureus*.	[134]
β-CyD	PLA	Cinnamon (CEO)	High antimicrobial activity due to released CEO	[135]
β-CyD	PVA	Cinnamon (CEO)	Stronger antimicrobial activity with incorporated lysozyme	[136]
β-CyD	Chitosan and PVA	Oregano and cinnamon EOs	Lower release of Oregano EO than CEO	[137]
β-CyD	PLA	Cinnamaldehyde (CA)	Higher release with increasing CA content	[138]
β-CyD	Zein	Eucalyptus EO	Higher antimicrobial activity with increasing EEO content	[140]
HP-β-CyD	PLA	Gallic acid	Increasing release rate with CyD incorporation	[141]
β-CyD	Pullulan	Perillaldehyde	Higher release with increasing humidity	[142]
α-CyD	Xanthan	Hexanal	CyD increased the release rate	[147]
γ-CyD	PLA	α-Tocopherol (α-TC)	CyD increased higher release of α-TC.	[148]
β-CyD	PAA	Quercetin	Nanofibers showed enhanced release rate than the films	[149]
β-CyD	PVA	Retinyl acetate (RA)	Slower release with CyD incorporation	[150]

**Table 2:** Overview of polyCyD/drug electrospun nanofibers used in drug delivery.

CyD Type	Polymer Additive	Active Molecule	Release Data	Ref
PolyCyD	PMAA	Propranolol hydrochloride (PROP)	40% Release (uniaxial PMAA:polyCyD (60:40, 80:20), 20% release from coaxial fibers	[151]
PolyCyD	PCL, PVP	Fluconazole	Burst release ((FLU-poly-α-CyD)-IC/PCL and (FLU-poly-β-CyD)-IC/PCL mats showed a burst of 85% in the first 15 min)	[152]
HP-β-CyD	Chitosan, citric acid (as cross-linker)	Triclosan	Higher release at lower pH (5.5), 80% release in 10 h	[153]
PolyCyD (peracetyl-β-CyD polymer)	-	Vitamin B2	60% (pH = 1.2) and 40% (pH = 7.4) release after 170 h	[154]
Poly aldehyde β-CyD (PA-β-CyD).	Gelatin	Chloramphenicol	Burst release for gelatin/drug (90% in 30 min), 90% release in 48 h for 7.5 and 10 wt.% PA-β-CyD	[155]
Poly-amino-β-CyD	PCL	Atorvastatin calcium trihydrate	TNF-α inhibition reached about 60% at 48 h (no dose effect), and up to 80% for IL-6, depending on the dose	[157]
Chitosan grafted carboxymethyl-β-CyD (CM β-CyD)	Chitosan	Salicylic acid	90% after 24 h at 37 °C, 84% after 24 h at 20 °C	[158]
Thiolated CyD	pEVOH/sH-CyD/PMDI	Vancomycin	Slow release	[159]
β-CyD	β-CyD/PMDA polymer	*N*,*N*-diethyl-3-toluamide (DEET)	Sustained release of all loaded DEET in 2 weeks.	[160]

**Table 3:** Overview of polymer-free CyD/drug ICs electrospun nanofibers used in drug delivery.

CyD type	Active molecule	Release data	Ref
HP-β-CyD, HP-γ-CyD	Triclosan	Rapid release on contact with water and significant inhibition against *E. coli* and *S. aureus*	[165]
HP-β-CyD, HP-γ-CyD	Camphor	In gas phase, faster release at higher temp., faster for the HP-β-CyD system	[166]
HP-β-CyD, HP-γ-CyD, and M-β-CyD	Eugenol	Rapid release on contact with water, enhanced antioxidant activity than eugenol itself	[167]
HP-β-CyD, HP-γ-CyD	Cineole and *p*-cymene	Rapid release along with the fiber dissolution	[168]
HP-β-CyD, HP-γ-CyD, and M-β-CyD	Linalool	Rapid release, significant inhibition against the growth of *E. coli* and *S. aureus*	[169]
HP-β-CyD, HP-γ-CyD and M-β-CyD	Limonene	25% Release M-β-CyD/limonene-IC-NF, 51% release HP-β-CyD/limonene-IC-NF, 88% release HP-γ-CyD/limonene-IC-NF in 100 days	[170]
HP-β-CyD, HP-γ-CyD and M-β-CyD	Geraniol	Long-term stability of geraniol in gas phase	[171]
HP-β-CyD, HP-γ-CyD and M-β-CyD	Thymol	Immediately on contact with water	[172]
HP-β-CyD, HP-γ-CyD	Menthol	Rapid release along with the fiber dissolution	[173]
HP-β-CyD, HP-γ-CyD and M-β-CyD	Vanillin	Immediately on contact with water, enhanced antioxidant activity with nanofibers	[174]
HP-β-CyD	Vitamin E	Rapid and enhanced release, higher antioxidant activity with CyD	[175]
HP-β-CyD, HP-β-CyD	Carvacrol	Rapid release on contact with water	[176]
SBE_7_-β-CyD	Sulfisoxazole	Rapid and enhanced release of sulfisoxazole on contact with water	[179]
HP-β-CyD	Diclofenac sodium	Release in few minutes	[180]
HP-β-CyD	Spironolactone	Total release in 1 h	[181]
Phenylacetic-β-CyD	Doxorubicin, fluorescein isothiocyanate-dextran (FITC-dextran), recombinant human insulin (FITC-labeled insulin) and chlorin e6	50% Release of drugs in vitro in 30 days, ~100% release of chlorin in vivo on day 28	[182]

## Abbreviations

Ac-β-CyDP	Peracetyl-β-CyD polymer
AITC	Allyl isocyanate
AV	Aloe Vera
CEO	Cinnamon essential oil
CIP	Ciprofloxacin
CUR	Curcumin
CyD	Cyclodextrins
CZ	Clotrimazole
DDS	Drug Delivery System
DPPH	2,2-Diphenyl-1-picrylhydrazyl
*E. coli*	*Escherichia coli*
EEO	Eucalyptus essential oil
EtOH	Ethanol
FDA	Federal Drug Administration
GC-MS	Gas chromatograph mass spectrometry
HCPT	Hydroxycamptothecin
HPC	Hydroxypropyl cellulose
HP-β-CyD	Hydroxypropyl-β-cyclodextrin
IC	Inclusion-Complex
MX	Meloxicam
M-β-CyD	Methyl-β-Cyclodextrin
NAP	Naproxen
PCL	Polycaprolactone
PEG	Poly(ethylene glycol)
PEO	Poly(ethylene oxide)
PLACL	Poly (l-lactic acid-*co*-ε-caprolactone)
PLLA	Poly (l-lactic acid)
PMAA	Poly(methylacrylic acid)
PolyCyD	Polycyclodextrin
PROP	Propranolol hydrochloride
PS	Polystyrene
PVA	Poly(vinyl alcohol)
RA	Retinyl acetate
RH	Relative humidity
*S. aureus*	*Staphylococcus aureus*
SA	Salicylic acid
SAg	Silver sulfadiazine
SFS	Sulfisoxazole
TAM	Tamoxifen
TCN	Tetracycline
VRC	Voriconazole
